# Development and validation of a practical solution for detecting motion artefacts in the EOS X-ray system

**DOI:** 10.1038/s41598-024-55373-2

**Published:** 2024-02-28

**Authors:** Vanessa Vallesi, Ganesh Shetty, Michael Moll, Peter Zweers, Markus Berger, Ernst Christiaanse, Masoomeh Pishgahi, Tobias Pötzel, Michael Fiechter, Giuseppe A. Zito, Rajeev K. Verma

**Affiliations:** 1 Department of Radiology, Swiss Paraplegic Centre, Nottwil, CH Switzerland; 2 Swiss Paraplegic Research, Nottwil, CH Switzerland; 3 Department of Spine Surgery, Swiss Paraplegic Center, Nottwil, CH Switzerland

**Keywords:** Musculoskeletal system, Translational research

## Abstract

The EOS™2D/3D system is a low-dose, 3D imaging system that utilizes two perpendicular X-ray beams to create simultaneous frontal and lateral images of the body. This is a useful modality to assess spinal pathologies. However, due to the slow imaging acquisition time up to 25 s, motion artifacts (MA) frequently occur. These artifacts may not be distinguishable from pathological findings, such as scoliosis, and may impair the diagnostic process. The aim of this study was to design a method to detect MA in EOS X-ray. We retrospectively analyzed EOS imaging from 40 patients wearing a radiopaque reference device during imaging. We drew a straight vertical line along the reference device. We measured deviations from it to quantify MA, presenting these findings through descriptive statistics. For a subset of patients with high MA, acquisitions were repeated after giving specific instructions to stand still. For these patients, we compared MA between the two acquisitions. In our study, a substantial proportion of patients exhibited MA ≥ 1 mm, with 80% in frontal projections and 87.9% in lateral projections. In the subjects who received a second acquisition, MA was significantly lower in the second images. Our method allows for a precise detection of MA on EOS images through a simple, yet reliable solution. Our method may improve the reliability of spine measurements, and reduce the risk of wrong diagnosis due to low imaging quality.

## Introduction

In conventional radiology, spinal deformities such as scoliosis are identified in anterior–posterior, hereafter referred to as frontal, and lateral projections on X-ray images. However, deformities of the spine are three-dimensional, and the longitudinal (i.e., axial) changes are not typically taken into account in conventional X-rays, although they are of clinical and therapeutic importance^1–3^. With its new X-ray detection technology, the EOS recording system (EOS imaging, Paris, France) allows for a 3D reconstruction and display of the entire spine^1–3^. The acquisition is performed in a normal standing or sitting position. The X-ray source moves simultaneously in both planes (frontal and lateral projection) from top to bottom, and the spinal column can be reconstructed in three dimensions using the post-processing software “sterEOS” (EOS Imaging, France). Furthermore, numerous quantitative parameters of the spine can be determined, such as the Cobb angle, sagittal vertical axis, pelvic tilt or sacral slope^1,3^. Various studies have shown that EOS imaging provides reliable and precise 3D reconstructions of the spine^4–7^, that cannot be achieved with conventional X-ray images. Moreover, EOS technology requires only a fraction of the radiation dose of conventional X-rays^1,4^, 5–10 times smaller. However, the recording time can take up to 25 s, which is significantly longer than 1–2 s of conventional X-ray^3^, and during this time, patients have to stand or sit motionless in the device. These scanning times are dependent on the patient's height, with taller patients generally requiring longer scanning times. This significantly increases the risk of motion artifacts. Indeed, motion artifacts are a well-known problem for this technology^8,9^. They have been described as unexpected but common, and are the direct result of the imaging method^8^. These artifacts can be easily detected in the long bones of the lower extremity, due to curvature of the bone, but might be difficult to detect in the spine^8^. In particular, motion artifacts in the spine can resemble other pathologies, such as scoliosis, and lead to misdiagnosis^9^. Research has attempted to reduce artifacts, for instance by shortening the scan time^10^, however, a definitive solution to the problem of motion artifacts has not been given yet, and in some cases, the use of EOS imaging has been discouraged in patients who are not able to stand still for the duration of the examination^11^.

In this study, we quantified motion artifacts and described a simple, yet reliable method to detect motion artifacts in EOS imaging. It consists of a hardware setup equipped with a reference device, a straight metal wire, attached to the patient's body. We assumed that any motion artifact equally affects the imaging of the reference device and the spine. We used the method to investigate: (i) the overall amplitude of motion artifacts in EOS imaging of the spine; (ii) differences in motion artifacts between first and second acquisition, for those patients who repeated the X-ray due to high movement; and (iii) correlation between motion artifacts and age. We hypothesize that patients requiring repeated measurements due to initial movement will exhibit reduced motion artifacts in their subsequent images, indicating the potential impact of patient positioning and instruction.

## Methods

### Design

**Figure 1:**
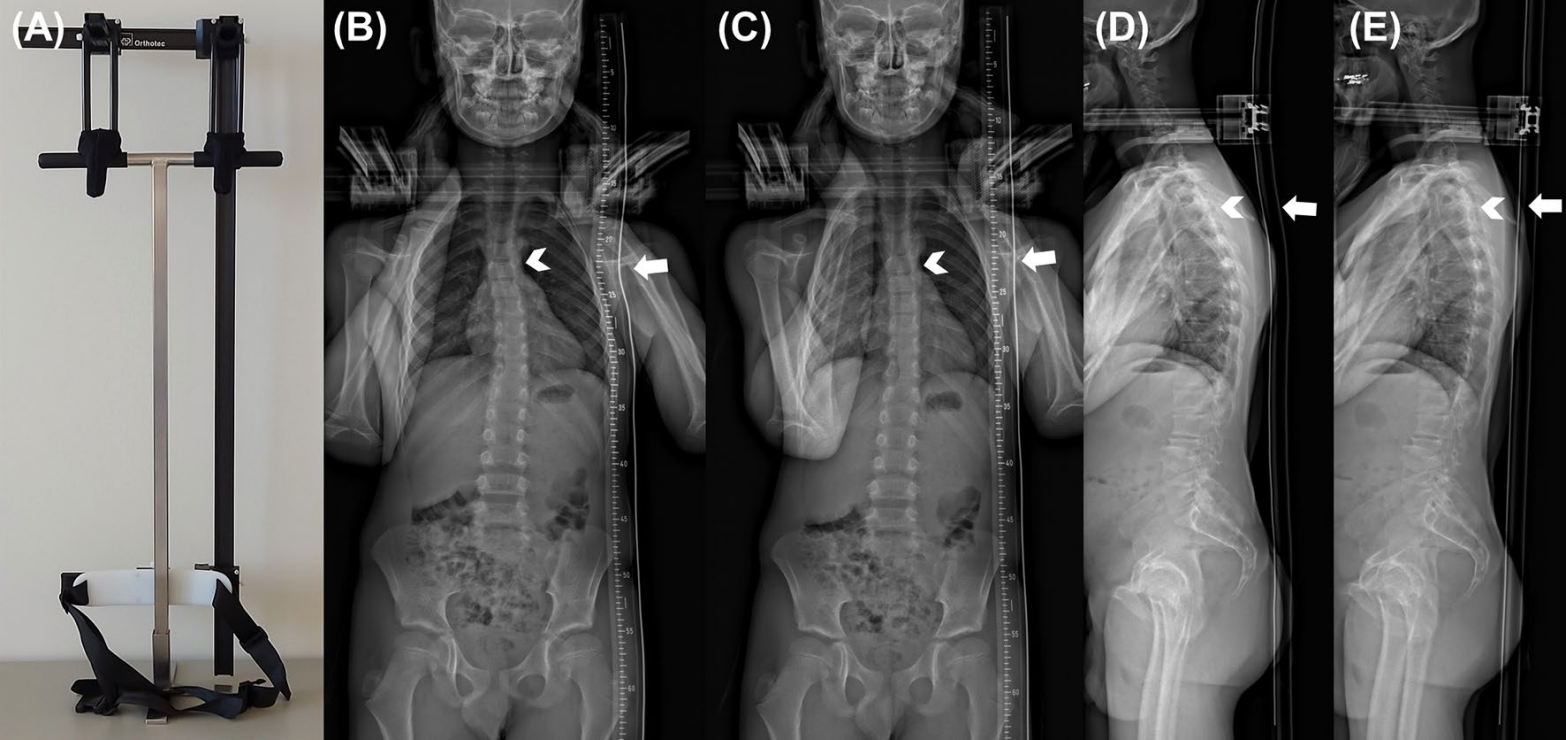
Hardware setup and X-ray images. (**A**) Picture of the developed setup with a metal wire as reference device. (**B**) image of a male 8-years old patient, where the metal wire is not straight (arrow), but bent at the height of the upper thoracic spine. These motion artifacts resemble a slight scoliosis at the level of Th5 / Th6 (arrow head). (**C**) image of the same patient after repeating the acquisition. The metal wire is straight (arrow) and no scoliotic change is found (arrow head). (**D**) image of a 40-years old male patient, where the metal wire is mildly bent (arrow) at the level of the upper thoracic spine, resembling a pronounced kyphosis (arrow head). (**E**) image of the same patient after repeating the acquisition. The metal wire is straight (arrow), and the upper thoracic spine is less kyphotic (arrow head).

We designed a straight metal wire, vertically oriented, that can be attached to the body of the patient as a reference device (Fig. 1A). The frame with the metal wire was developed and constructed by the Orthotec (Swiss Paraplegic Society, Nottwil, Switzerland) under the guidance of the radiology specialist and the radiologist. The reference device is 115 cm long, with a straight radiopaque wire fixed into the plastic (polycarbonate, carbon and teflon). The reference device is fixed to the body of the patient by means of height and laterally adjustable brackets, positioned at a 90° angle. Shoulder pads on a fixed frame are attached at a height of about 15 cm in order not to cover the relevant areas in the cervical spine and to avoid overlapping artifacts. In the lower part of the wire, a height-adjustable bracket was also attached, which was fixed to the patient in the area below the greater trochanter with a belt. This was done to fix the measuring wire as close to the patient as possible. The shoulder padding was made from durable, washable material (nylon/synthetic fiber). Except the metal wire, no metallic materials were used in order to avoid artifacts.

### Patients and data acquisition

The study was conducted as a retrospective analysis of data acquired at the Department of Radiology of the Swiss Paraplegic Centre (Nottwil, Switzerland) between September 2021 and December 2021 as part of the standard clinical routine for the EOS acquisition system. All methods were performed in accordance with the relevant guidelines and regulations, and aligned with the latest version of the Declaration of Helsinki. The study was approved by the Ethics Committee of the North-West and Central Switzerland (EKNZ study 2022–01097). The Ethics Committee waived the collection of informed consent because, due to the retrospective study design, it was not possible to contact all patients, and the interests of the research outweighed the interests of the persons concerned.

We analyzed 40 consecutive patients who fulfilled the following inclusion criterion: Patients who could stand during acquisition of the whole spine and the pelvis. For all patients, the reference device was attached to their body (Fig. 1B–E), preferably in frontal and lateral projection, or in at least in one of them. The mean scan exposure time was 10.3 ± 2.1 s, the mean dose area product was 3.77 ± 0.9 Gy cm^2^, and the mean kilovoltage was 96.03 ± 5.8 kV.

### Identification of motion artifacts

**Figure 2:**
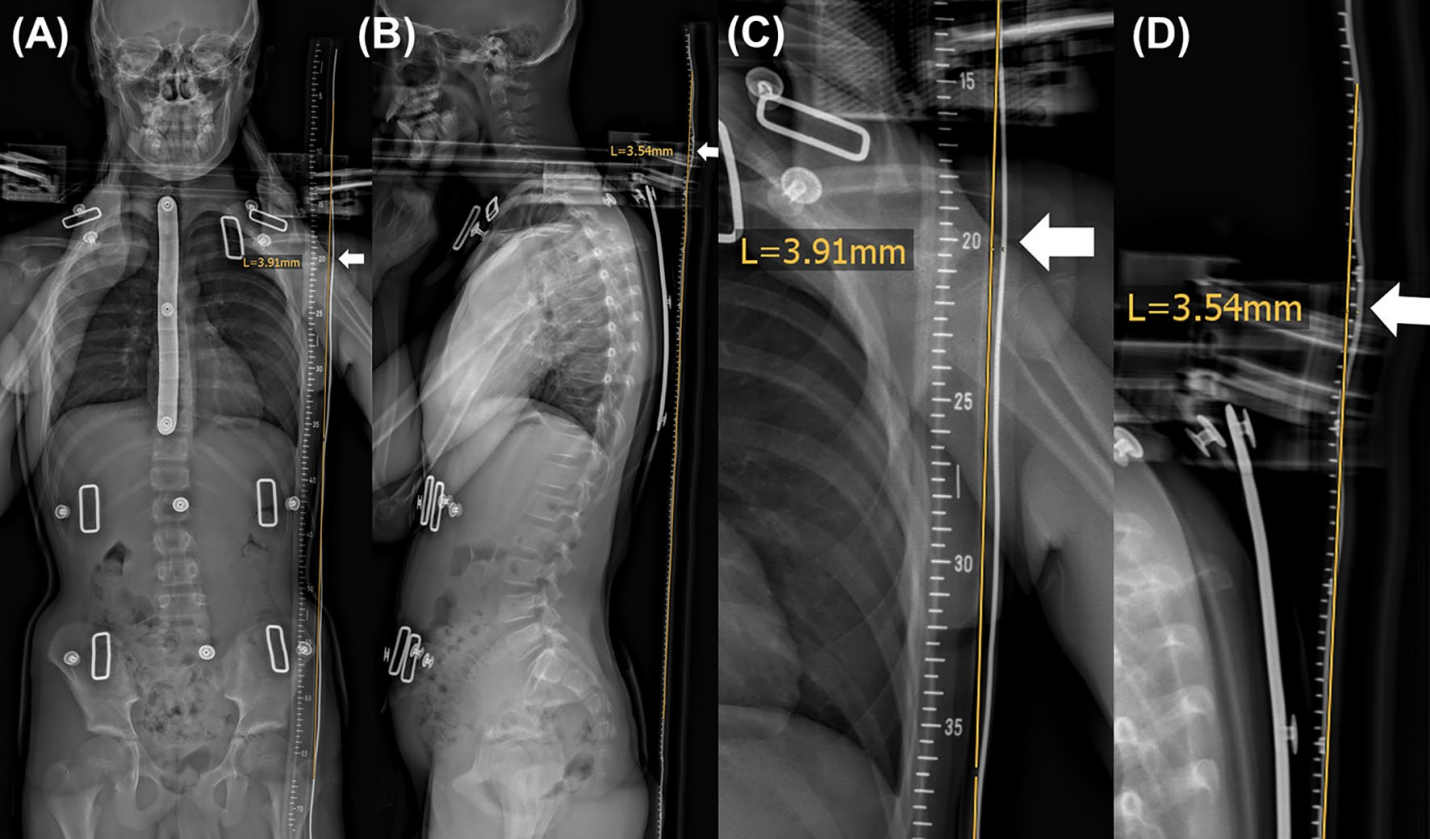
Measurement of motion artifacts in a 11-years old male child with a corset. (**A**) frontal projection. (**B**) lateral projection. (**C**) zoomed-in image of frontal projection. (**D**) zoomed-in image of lateral projection. The straight line was drawn on the reference device between the levels C2 and S3.

Measurements were carried out on a PACS workstation by two radiologists (RKV and GS) with 22 and 9 years of experience, respectively. A line was drawn on the image exactly on the reference device, from the level of the second cervical vertebral body to the level of the third sacral vertebral body (Fig. 2).

Motion artifacts were defined as the deviation between the reference device and the ideal straight line. If the reference device was imaged as straight as in reality, no movement artifact was considered (Fig. 2), conversely, in case of deviation from the straight line, the region with the largest horizontal shift between the metal wire and the drawn line was identified and measured in millimeters.

In addition, the level of the deviation was noted according to five regions in the spine: (1) cervical spine (cervical vertebral bodies 1–7), (2) upper thoracic spine (thoracic vertebral bodies 1–6), (3) lower thoracic spine (thoracic vertebral bodies 7–12), (4) lumbar spine (lumbar vertebral bodies 1–5), and (5) sacral spine (sacral vertebral bodies 1–5). Further, the direction of motion was analyzed, i.e., movement to the right or to the left in the frontal projection, and ventral or dorsal movement in the lateral projection (Fig. 2).

Moreover, we identified the patients who received a second X-ray of the spine due to high motion artifacts in the first acquisition, and analyzed both acquisitions as described above.

### Statistical analysis

We first applied the Shapiro–Wilk normality test to check for the normal distribution. Since the motion artifacts showed non-normal distributions, non-parametric tests were used for all analyses. We performed the following analyses:

1. We described the amplitude of the motion artifacts between the reference device and the corresponding straight line on the images, in both frontal and lateral projection, respectively, with central tendency and variability parameters.
2. For the patients who received a second X-ray, we compared the amplitude of the motion artifacts in mm between measurements of the first and the second image with an Exact Wilcoxon Signed-rank test.
3. We calculated the correlation between motion artifacts in mm and age, in both frontal and lateral projections, with the Spearman’s Rank Correlation.

For all statistical tests, the 95% confidence intervals were included. All statistical models and graphs were created with the statistical software R studio^12^, the R packages rstatix^13^ and tidyverse^14^ were used.

## Results

A total of 40 patients fulfilled the inclusion criteria and were included in the study (26 male, 14 female; mean age = 49.8 ± 22.6 years). All 40 patients were included in the analysis of the frontal projection. Seven patients were excluded from the lateral projection analysis, since the reference device was only partially visible on the images due to e.g., osteoporosis or distinct adiposities.

Six patients repeated the acquisition (six male, mean age = 35.5 ± 24.9 years), due to distinct motion artifacts. All six patients were included in the analysis of the frontal projection, one patient was excluded from the lateral projection analysis, due to a partially visible reference device on the image. No significant difference in motion artifact measured in mm was found when comparing female vs. male (Z = 0.73 and p-value = 0.465 in the frontal projection, Z = 0.26 and p-value = 0.795 in the lateral projection).

### Occurrence and Amplitude of Motion Artifacts

**Figure 3:**
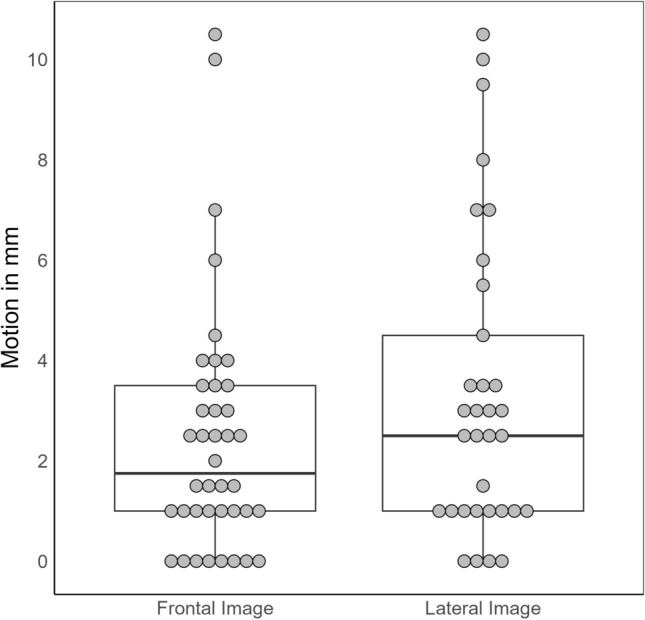
Boxplot of the movement artifacts, in the frontal and lateral projections.

Results indicated that motion artifacts in the frontal projection had a median of 1.75 mm and an interquartile range (IQR) of 2.5 mm, while in the lateral projection, the median was 2.5 mm with an IQR of 3.5 mm. Additionally, 80% (32 out of 40) of the patients exhibited motion artifacts ≥ 1 mm in the frontal projection, and 87.9% (29 out of 33) in the lateral projection, with a maximum motion artifact of 10.5 mm in both directions. For further details see Fig. 3.

Most of the motion artifacts in the frontal projection occurred at the level of the lower and upper thoracal spine with 37.5% (lower spine), and 30% (upper spine), respectively. Some patients showed motion artifacts at cervical, lumbar or sacral level (Table 1). Further, on the frontal view, 45% of patients moved to the right direction, 35% to the left. On the lateral view 54.5% of the detected artifacts were at the level of the lower thoracal spine, whilst the other spine levels showed noticeably less motion artifacts (Table 1). 54.4% of the patients moved in the anterior direction, whilst 33.3% moved in the posterior direction.

**Table 1:** Occurrence of motion artifacts (i.e., number of patients) at different levels of the spine.

Categories	No motion artifacts	Cervical spine (C1–C7)	Upper thoracic spine (T1–T6)	Lower thoracic spine (T7–T12)	Lumbar spine	Sacral spine
MA frontal view	8	1	12	15	2	2
MA lateral view	4	2	3	18	5	1

Only the regions with the strongest deviation are listed.

### Comparison of the first and the second image in those patients with repeated, second imaging

**Figure 4:**
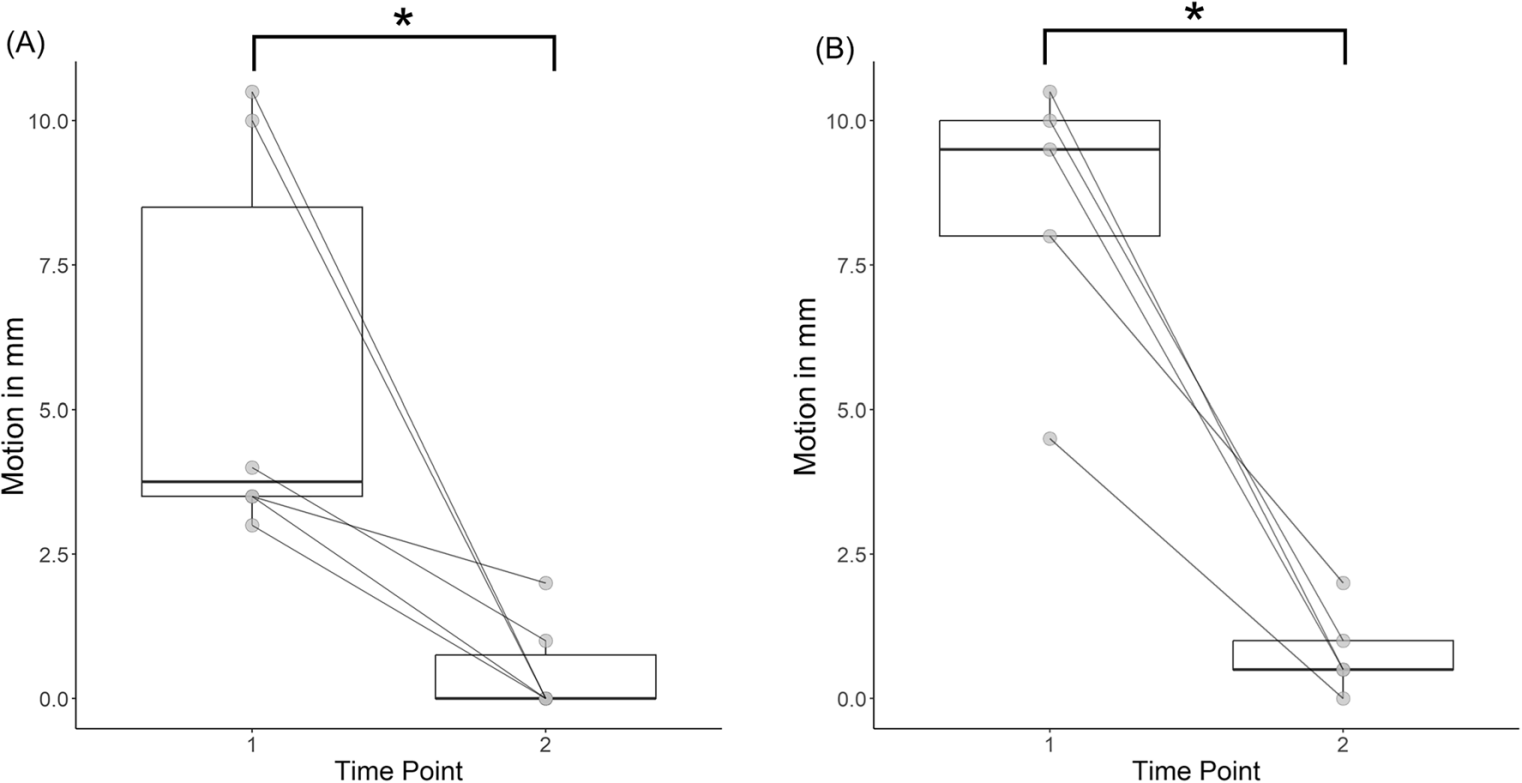
Results of the comparison between first and second acquisition. (**A**) Frontal projection. (**B**) Lateral projection. *Depicts significant differences at an Exact Wilcoxon Signed-rank test. Each line represents one patient and the level S3. Horizontal deviation between the wire and the line were measured in mm and noted (arrows), at the level of the largest deviation.

For the six patients who showed high motion artifacts, the median deviation was 3.75 ± 5.00 mm in the frontal projection, and 9.50 ± 2.00 mm in lateral one. After repeating the acquisition a second time and instructing patients thoroughly on remaining still for the entire duration of the acquisition, the motion was significantly reduced in the frontal projection, whereas a trend towards significance was observed in the lateral projection (V = 21.00, p-value = 0.031, 95% CI [1.5, 10.5], estimated sample median = 3.5 mm in the frontal projection, V = 15.00, p-value = 0.063, 95% CI [4.5, 10.0], estimated sample median = 7.5 mm in the lateral projection) (Fig. 4).

### Correlational analysis of motion artifacts with age

The correlational analysis showed no significant correlation between motion artifacts and age (Spearman’s rho = − 0.26, p = 0.11 in the frontal projection, Spearman’s rho = − 0.26, p = 0.15 in the lateral projection).

## Discussion

The EOS imaging system is a very helpful method to acquire images of the whole spine in three dimensions with a fraction of the radiation dose needed with the conventional X-ray technique.

Motion artifacts in EOS imaging acquisition can be a major issue^1,8,9,15–18^. It has been suggested that patients who are not able to stand still for the duration of the examination are not suitable for an examination with EOS, and they should be examined with other modalities, such as conventional X-ray or computer tomography^11^. In our study, we observed that a substantial proportion of our sample exhibited motion artifacts greater than 1 mm, with 80% of the patients in the frontal projection and 87.9% in the lateral projection. These motion artifacts were effectively identified by attaching a vertical metal wire, serving as a reference device, to the patient’s body. Further, the extent and location of motion artifacts could be exactly determined. Our study showed that, although we instructed patients to stand still during acquisition, 80% had motion artifacts. This is in line with existing literature, where motion artifacts have been regularly reported^15^. In our study, the majority of motion artifacts was negligible, but in 15% of the subjects, a repetition of EOS imaging was needed.

Further we found distinctly more motion artifacts. This is presumably because our attached metal wire allowed us to detect the smallest motion artifacts, that are hard to see on normal images (see Fig. 2 as example).

To the best of our knowledge, only one study analyzed motion artifacts in EOS imaging, with a setup partially comparable to ours, i.e., a metal wire of the spinal fusion was used to detect motion artifacts in the form of angular deviations^15^. As in our study, the authors observed frequent occurrence of artifacts, however, they did not affect radiographic measures after spinal instrumentation. A reason for these results might be that the regular observation of artifacts led to practical experience in acquiring EOS images, for instance by setting acquisition parameters and carefully instructing patients.

Based on our experience with the EOS system, measurement values can be severely distorted by movement artifacts, which might lead to incorrect diagnosis of e.g., scoliosis, or incorrect curvature of the spine. This may result in incorrect therapy recommendations. Although in our cases motion artifacts were likely not leading to incorrect therapy recommendations, repeated images were performed to safely avoid them.

To our knowledge there are no studies yet that describe the extent and region of motion artifacts. The only study addressing motion artifacts in EOS imaging measured spine angles in degrees^15^, but the extent in millimeters and location has never been addressed. Our study revealed that the thoracic spine, especially the lower part, is the location where most motion artifacts appear, both in frontal and lateral projections. We assume that the thoracic portion of the spine is the part that moves the most, especially when standing restless for up to 25 s, hence its imaging is the most susceptible to motion artifacts. Of note, the thoracic spine is the region where idiopathic scoliosis typically occurs^19–21^. It is therefore of utmost importance that motion artifacts are correctly identified in this region, and that the risk for misdiagnosis is reduced.

Slight motion artifacts can be expected, since acquisition time can last for many seconds. It might be difficult, e.g. for children or ill persons to stand still for that period. Although motion artifacts are described regularly in children^8,15^, we found no significant correlation between age and motion artifacts. However, our sample included only five participants under the age of 18 years, which limits the generalizability of our findings. Despite this limitation, our result demonstrates that motion artifacts were present across all age groups. Moreover, distinct motion artifacts observed in some subjects required repeat acquisitions. Although these subjects represented only 15% of the entire study population, our results highlighted two important aspects: (i) the potential role of proper instructions in reducing motion artifacts. Our comparison of scans taken before and after providing additional instructions suggests this as a hypothesis worth further exploration. However, confirming this would require a prospective controlled study design; and (ii) the current clinical practice needs a standardized, reliable, and reproducible method to check images for motion artifacts. More specifically, the decision to repeat the image acquisition in our subsample was driven by the high motion artifacts identified with our method. This is particularly relevant when considering the additional radiation dose due to a second exposure to X-rays. Even though the EOS system needs only a fraction of the radiation dose that conventional X-ray acquisition systems need, the indication for a repetition of imaging should be handled with care. We believe that the use of a reference device allows clinicians to safely rule out the contribution of motion artifacts, and thus to prescribe a second exposure to X-ray only when necessary.

A limitation of our study is that there is no previous work that quantified motion artifacts in millimeters in EOS imaging. Moreover, the reference device we used can overlay parts of the spine. We designed our frame so that the metal wire does not cover important parts of the spine, and our results showed that most of the spine is normally pictured, with only the lower part of the cervical spine partially covered. Depending on the clinical question, this overlay is tolerable for most of the indications where EOS imaging technique is applied. Another fact to consider is that the second generation of EOS imaging systems (EOSedge, EOS Imaging, France) needs less acquisition time than the first generation EOS System described in this study. It is possible that, by reducing the acquisition time, the motion artifacts will be also smaller. New investigations of the new system will be needed to evaluate whether a control of motion artifacts is required or not.

In our study, we only focused on evaluating the EOS imaging system. However, it is plausible that motion artifacts might be a common challenge in various slot-scanning X-ray systems. This issue could potentially arise in other types as well, suggesting a broader applicability of our findings across different X-ray imaging technologies. In conclusion, our study revealed that attaching a straight metal wire to the subject`s body enables the reliable detection and quantification of spinal motion artifacts, including their amplitude and location. Even in case of minor motion artifacts not leading to incorrect diagnosis, the referring physician gets an accurate estimation of their extent, and can take them into account during the clinical evaluation. We recommend to apply this method to those patients where motion artifacts are likely to occur, e.g., patients with standing or gait problems. This is a cost-effective and easy-to-implement solution. For future studies, a more sustainable approach would be the development of an additional automatic, software-based correction, which would help avoid the need for multiple scans.

## Data Availability

The data that support the findings of this study are available from the corresponding author upon reasonable request.
